# Navigating the Panzootic Era of HPAI H5N1: Bridging Surveillance and Countermeasure Deficits

**DOI:** 10.3390/v18070757

**Published:** 2026-07-10

**Authors:** Daniel R. Perez

**Affiliations:** Department of Population Health and Center for Vaccines and Immunology, College of Veterinary Medicine, University of Georgia, Athens, GA 30602, USA; dperez1@uga.edu

The evolutionary trajectory of highly pathogenic avian influenza (HPAI) H5N1 has fundamentally shifted from a sporadic agricultural pathogen to an enduring global panzootic. Since the emergence of the 2.3.4.4b clade in late 2020, the virus has transcended its traditional localized agricultural disruptions to establish endemic circulation within wild bird reservoirs across all inhabited continents, including recent, unprecedented incursions into the sub-Antarctic and Antarctic regions as well as Oceania. This dramatic expansion in host plasticity has enabled the virus to infect over seventy distinct mammalian species, triggering catastrophic mortality events in marine mammals across South America and widespread, unprecedented outbreaks within commercial dairy cattle herds in the United States. Consequently, the risk of zoonotic spillover has escalated, exposing humans to novel transmission pathways through complex multi-species interfaces such as contaminated milking equipment, unpasteurized dairy products, and infected companion animals.

Despite these alarming epidemiological developments, critical gaps in our collective knowledge and structural preparedness continue to hinder global response efforts. A profound chasm exists in understanding the ecological drivers of spillover, particularly how massive habitat fragmentation, ecological compression, and the stark contrast between unchanged backyard farming and high-density industrial agriculture fuel viral reassortment. Furthermore, there is a dangerous systemic vulnerability in the global epidemiological response due to an over-reliance on passive surveillance frameworks, which are inherently retrospective and systematically fail to capture asymptomatic viral shedding, subclinical infections, and the insidious circulation of low-pathogenic precursors. This reliance creates vast geographic blind spots, particularly in resource-limited regions across South America, the Middle East, and Africa, allowing the virus to silently evolve and adapt to mammalian physiology entirely undetected until a major spillover event occurs.

This Special Issue curates groundbreaking research that directly confronts these systemic vulnerabilities through advanced genomics, ecological tracking, and the evaluation of next-generation countermeasures. Through meticulous phylogeographic modeling and Bayesian analysis, the featured studies expose geographic surveillance deficits in the Middle East and South America, charting the precise molecular drifts and host adaptations driving the virus’s southward expansion. The issue further bridges the clinical and ecological divides by analyzing the severe cascading impacts on apex predators acting as environmental sentinels, the rising vulnerability of companion animals serving as viral mixing vessels, and the devastating clinical realities of H5N1 infection during human pregnancy, which carries a staggering maternal case-fatality rate exceeding 90%. Crucially, the curated research showcases rapid, adaptable genomic workflows deployed during the U.S. dairy cattle outbreaks to overcome diagnostic hurdles, and it successfully validates highly efficacious, scalable DNA vaccine platforms (such as pVAX-H5) that bypass the sluggish production timelines of traditional egg-based manufacturing.

As HPAI H5N1 continues to permanently breach historical ecological boundaries and embed itself within complex multi-host networks, future research must forcefully pivot toward proactive and highly scalable mitigation strategies. While the accelerated development of next-generation nucleic acid vaccines represents a critical step for human pandemic preparedness, safeguarding the vast agricultural interface demands innovative, large-scale delivery mechanisms for animal populations. Therefore, scientific inquiry must primarily focus on the development of advanced vaccination strategies that allow for seamless mass applications via drinking water, feed formulations, or other alternative non-invasive mass vaccination methods. Integrating these highly efficient mucosal or oral delivery systems into standard industrialized and traditional agricultural practices will be absolutely paramount in severing transmission chains, proactively reducing environmental viral shedding, and ultimately achieving global resilience against the panzootic threat.

